# Predicting Corn Moisture Content in Continuous Drying Systems Using LSTM Neural Networks

**DOI:** 10.3390/foods14061051

**Published:** 2025-03-19

**Authors:** Marko Simonič, Mirko Ficko, Simon Klančnik

**Affiliations:** Faculty of Mechanical Engineering, University of Maribor, Smetanova 17, SI-2000 Maribor, Slovenia; mirko.ficko@um.si (M.F.); simon.klancnik@um.si (S.K.)

**Keywords:** drying, moisture prediction, big data, artificial intelligence, LSTM

## Abstract

As we move toward Agriculture 4.0, there is increasing attention and pressure on the productivity of food production and processing. Optimizing efficiency in critical food processes such as corn drying is essential for long-term storage and economic viability. By using innovative technologies such as machine learning, neural networks, and LSTM modeling, a predictive model was implemented for past data that include various drying parameters and weather conditions. As the data collection of 3826 samples was not originally intended as a dataset for predictive models, various imputation techniques were used to ensure integrity. The model was implemented on the imputed data using a multilayer neural network consisting of an LSTM layer and three dense layers. Its performance was evaluated using four objective metrics and achieved an RMSE of 0.645, an MSE of 0.416, an MAE of 0.352, and a MAPE of 2.555, demonstrating high predictive accuracy. Based on the results and visualization, it was concluded that the proposed model could be a useful tool for predicting the moisture content at the outlets of continuous drying systems. The research results contribute to the further development of sustainable continuous drying techniques and demonstrate the potential of a data-driven approach to improve process efficiency. This method focuses on reducing energy consumption, improving product quality, and increasing the economic profitability of food processing.

## 1. Introduction

In the era of Industry 4.0, significant advances in digital technology have reshaped industrial sectors such as manufacturing [1], logistics [2], aerospace [3], energy [4], pharmaceuticals [5], and many others [6]. Agriculture 4.0 has emerged as a transformative force in agricultural practice, harnessing the principles and technologies of Industry 4.0 [7]. While there is no exact date that marks the beginning of Agriculture 4.0, the development gained interest in the late 2010s and early 2020s, along with the formalization of the term Industry 4.0 [8]. Building on this, Agriculture 4.0 represents a fundamental shift in various agricultural practices driven by technological innovation and data-driven decision making [9]. The growing world population and the demand for food make Agriculture 4.0 necessary in order to meet these challenges efficiently [10]. In the context of corn production and processing, various studies have investigated different aspects of optimization techniques in the field of genetics [11], yields [12,13], and post-processing [14].

In connection with the optimization of drying processes, a numerical and experimental study was carried out on high-frequency-assisted convective drying of corn as an alternative to traditional convective drying [15]. The effects of the tempering time and hot-air drying temperature on the properties of corn, particularly on starch, were also investigated. The investigations showed that a higher drying temperature had a decreasing effect on the starch yield and other properties, such as transparency and solubility. An adjusted tempering time resulted in starch with improved whiteness, solubility, and swelling capacity [16]. Using seed germination as an example, experimental data were collected and analyzed to investigate the influence of residual pressure in a peri-seed environment [17].

Artificial intelligence has proven its usefulness in numerous studies on drying processes. A control method using artificial intelligence for a continuous grain drying process was investigated, combining the accumulated temperature mechanism and a data-driven approach, taking into account the significant delay, nonlinearity, and parameter uncertainty inherent in the grain drying process [18]. In a study, the effects of high-power ultrasound on a laboratory-scale fluidized bed dryer for shelled corn were analyzed using artificial neural networks. Models were developed to predict the drying time, energy consumption, changes in moisture content, and quality indicators such as color and shrinkage [19]. Moisture content modeling using static and dynamic artificial neural networks showed promising results using a microwave hot-air dryer as an example for mushroom drying [20]. In addition to neural networks, the combination with genetic algorithms in the optimization of process parameters for fluidized bed drying has also proven to be promising. The hybrid approach predicted optimal conditions that led to the high-quality production of green tea within certain limits [21]. As part of the energy and exergy analysis, a study was carried out to investigate the hot-air drying of mushroom slices. This revealed a significant influence of air velocity and temperature on the energy and exergy parameters. Artificial neural network models were found to be effective in predicting system performance with satisfactory accuracy [22]. Similarly, artificial neural networks have proven to be useful for analyzing energy and exergy in the drying of potato cubes [23]. When analyzing sequential data, traditional neural networks can encounter challenges as they are unable to effectively capture the temporal dependencies contained in the data [24]. Utilizing the capabilities of recurrent neural networks, an intelligent control system for grain dryers using the example of rice drying was developed. The efficiency of backpropagation through time in managing complex nonlinear processes, such as grain drying, has been demonstrated [25]. In a study focusing on Pu-Erh tea during the sun-drying process, the GRU (gated recurrent unit) has also shown promising results [26]. Although backpropagation through time (BPTT) has proven useful in modeling grain drying, it is known to suffer from the vanishing gradient problem. Therefore, the long short-term memory (LSTM) architecture has been used in several other studies, as it is better able to capture long-term dependencies in sequential data [27]. Therefore, LSTM has been used as an effective approach for evaluating the moisture content of carrot slices during drying [28]. In addition, a deep learning model (LSTM) was used for the predictive control of an industrial multistage countercurrent drying process for rice [29]. A data-driven approach has been shown to be useful to effectively control a complex corn-to-sugar conversion process through the use of LSTM models [30]. In an example of drying potato slices, a multiple-input single-output (MI-SO) architecture was used to predict a single output (moisture content) using LSTM networks [31].

As mentioned in the previous sections, Agriculture 4.0 has already led to significant progress in the optimization of drying techniques. The efficiency, quality, and sustainability of the process are the key areas of these changes. Artificial intelligence has played a crucial role in optimizing various drying techniques for a wide range of products. Recurrent neural networks have been used extensively in this area through various data-driven approaches. LSTM models have also proven to be valuable tools in optimizing drying techniques for various grains, excluding corn, and, of course, in other agricultural applications. To address the gap in LSTM modeling using a data-driven example for corn drying modeling, this paper explores a data-driven approach for continuous corn drying using recurrent LSTM neural networks for outlet corn moisture content. Since data collection was human-dependent in the past, this research also delves into suitable methods for data imputation in the learning base and compares the results of learning on imputed data with learning on an alternative dataset. Since the LSTM approach for corn moisture prediction is new, different combinations of learning hyperparameters were also evaluated for corn moisture prediction at the output. By using this approach, the aim is to optimize the drying process time, temperatures, and energy efficiency.

We therefore strive to achieve the following research objectives:

1. The development and implementation of an LSTM recurrent neural network model to predict the moisture content of corn at the outlet of a continuous corn drying process.

2. The evaluation of the developed models using objective metrics, such as mean absolute error (MAE), mean square error (MSE), root mean square error (RMSE), and mean absolute percentage error (MAPE).

3. Visualization of the results using the example of moving averages and actual samples and predictions within the selected interval.

This paper is organized as follows: Section 2 provides information on data collection and sampling; Section 3 provides the numerical and visual results of the study; and Section 4 discusses the results, concludes with the research contributions, and provides further directions.

## 2. Materials and Methods

### 2.1. An Experimental Approach

In 2015, a continuous convective dryer (Bühler Group, Uzwil, Switzerland) was put into operation in the north-eastern part of Slovenia. Optimizing the process proved challenging during the dryer’s first harvest season. Therefore, an experimental approach was introduced to make the process more cost-efficient and sustainable. The main challenge was to predict the outlet moisture content of the corn at the exit based on the temperature process parameters.

The drying process runs in a continuous flow, with a material flow between 9 and 12 t/h, as specified in the dryer guidelines. The flow rate depends on the discharge interval, which is normally set between 120 and 160 s based on the moisture content in the inlet. As can be seen from the statistical representation of the dataset (Table 1), the average discharge interval was recorded at 153.2 s, which corresponds to an estimated average flow rate of 9.4 t/h.

**Figure 1:**
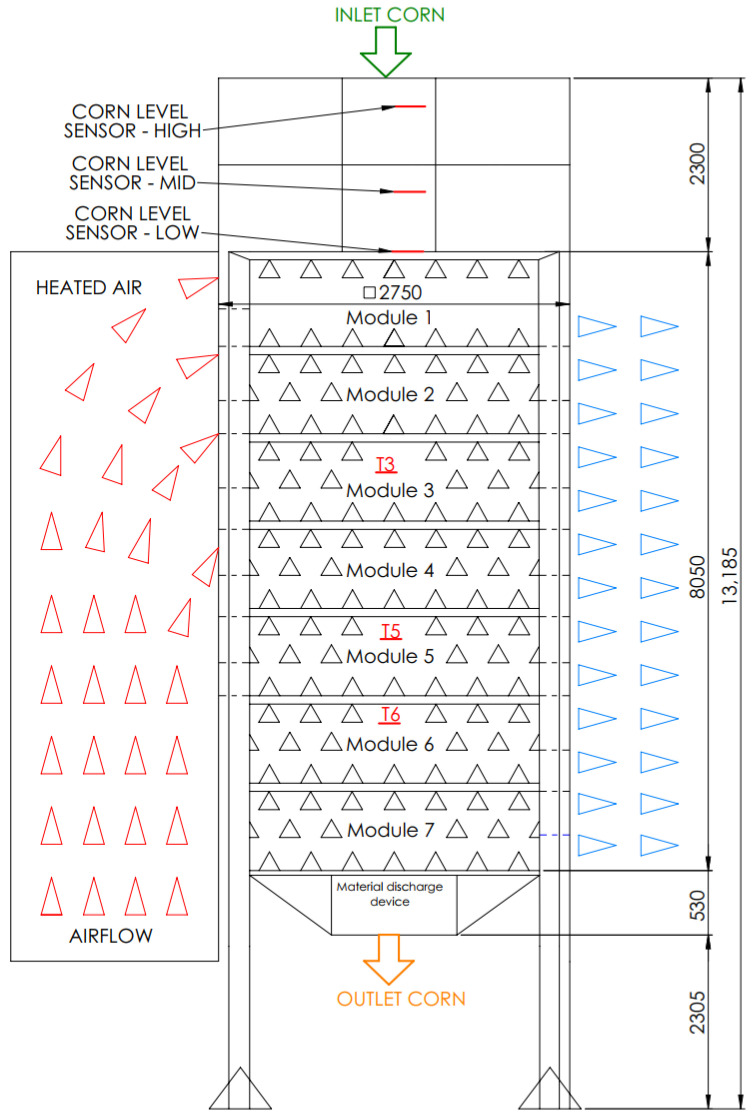
Drying tower schematics.

The drying tower has a total height of 13,185 mm and a square cross-section with a width and depth of 2750 mm, as can be seen in Figure 1. The drying silo, in which the actual drying process takes place, has a height of 8050 mm. The remaining height consists of the inlet area, which measures 2300 mm, and the material outlet area, which is 530 mm. In addition, the height of the drying tower includes a section of 2305 mm dedicated to the corn outlet transportation system.

The fresh corn enters the system at the top of the drying tower, moves vertically and continuously downward through the modules, and exits at the bottom. Each module contains internal baffles, shown as black triangles in Figure 1, which mix the corn as it flows downward. These baffles ensure the even distribution of the corn bed and improve heat transfer by constantly exposing new surfaces of the corn to the heated airflow, promoting more uniform drying throughout the process. The heated air enters the system on the left side of the dryer (red triangles in Figure 1), while the cooler air leaves the dryer on the right side (blue triangles). To monitor drying efficiency, a moisture sample is taken at the inlet—above the entrance—to determine the initial moisture content of the corn. Another sample is taken at the exit after the corn has left the system to measure the final moisture content and ensure that the drying process meets the desired specifications.

As the drying system works in a continuous flow, moisture measurements in the intermediate stages of drying are not possible. However, the drying behavior over time is reflected by variations in the module temperatures (*T*_3_, *T*_5_, *T*_6_) and the residence time of the material, which is regulated by the discharge interval (*D_I_*). These factors define the drying profile within the system and are taken into account in the LSTM model to capture time dependencies.

The material discharge device controls the discharge interval and thus directly regulates the corn throughput and drying efficiency. During unloading, the unloading device opens the hatch at the bottom of the dryer (seen in Figure 1) for 1.5 s and releases a controlled amount of dried corn before closing it and continuing the drying process. The temperature process parameters in the modules of the drying system are controlled by a computer control system with two main variables:

- The target temperature of the heated air *(T_A_)*, which determines the level of heat input during the drying process. It is controlled by the burner system, which adjusts the drying temperature to increase or decrease the intensity of moisture removal. A higher target heating air temperature speeds up the drying process but can lead to over-drying or damage to the grains, while a lower temperature can lead to insufficient moisture removal.
- The material discharge interval *(D_I_)*, which determines how long the corn remains in the drying system before it is discharged to the next stage. It regulates the retention time of the material in the drying modules and, thus, its exposure to heat. A shorter discharge interval increases the material flow but can lead to insufficient drying, while a longer interval enables a longer drying time but can reduce efficiency. The discharge interval is usually set based on the moisture content of the corn in conjunction with the target air temperature—with a higher air temperature allowing a shorter drying time—to achieve optimal drying performance.

To achieve efficient and uniform drying, it is crucial to find an optimal combination of these two independent variables. A balanced setting ensures that the drying process remains energy-efficient while maintaining the desired moisture content of the corn, minimizing the risk of over- or under-drying. Table 1 summarizes the conditions for the drying process.

If the moisture content of the corn does not reach the desired level, specific adjustments are made to optimize the drying process:

- Increased target air temperature (*T_A_*): A higher drying temperature accelerates the heating of the module much faster than an adjustment in the material discharge interval (*D_I_*). Although the effect is not immediate, *T_A_* changes have a faster impact than *D_I_* adjustments as the increased heat spreads through the system. In practice, *T_A_* is adjusted more frequently, as operators strive to maintain a constant process speed. However, *T_A_* is limited to a maximum of 120 °C to prevent grain damage and maintain product quality.
- Extended material discharge interval (*D_I_*): While *D_I_* adjustments take longer to impact the drying process, they are used when further moisture reduction beyond the *T_A_* limit of 120 °C is required. As higher temperatures and rapid drying can have a negative impact on material quality, *D_I_* is generally adjusted less frequently than *T_A_*.

The positions of the drying modules within the drying system are also shown in Figure 1. In addition to these modules, other continuous features of the drying process can be observed. At the top of the drying tower, the entrance for fresh corn is marked with a green arrow. Three corn level sensors, which are used to automatically regulate corn storage before drying, are also marked:

- Corn level sensor—High: Stops corn transport when the storage reaches maximum capacity (40 tons of corn).
- Corn level sensor—Mid: Triggers the automatic transport of corn into the dryer, which remains active until the material reaches the level sensor—High.
- Level sensor—Low: Stops the drying process due to lack of material and initiates automatic cooling of the dryer.

As the corn travels through the drying modules, various temperature sensors measure the temperatures of the material in the modules, which are marked in red as *T*_3_, *T*_5_, and *T*_6_. At the bottom of the diagram, the outlet corn is marked with an orange arrow indicating where the dried corn leaves the system. From there, the dried corn is transported to the storage silo and cooled to allow for possible long-term storage.

Originally, the experimental approach was analog, with operators relying on pen and paper to record data and predict moisture outcomes based on temperature process parameters in drying modules 5 and 6. As this method proved useful for predicting the outlet moisture content of corn, an additional temperature sensor was installed in drying module 3 to improve monitoring.

The operators continued to record data and predict moisture based on their experience until 2022. This persistence was crucial for the accumulation of an extensive dataset comprising 3826 samples. The data collected include the following variables structured in columns:

- Inlet corn moisture (*M_I_*);
- Target air temperature (*T_A_*);
- Material discharge interval (*D*_I_);
- Temperatures of the drying modules (*T*_3_, *T*_5_, and *T*_6_);
- Target variable: Outlet corn moisture (*M_O_*).

### 2.2. Moisture Sampling

**Figure 2:**
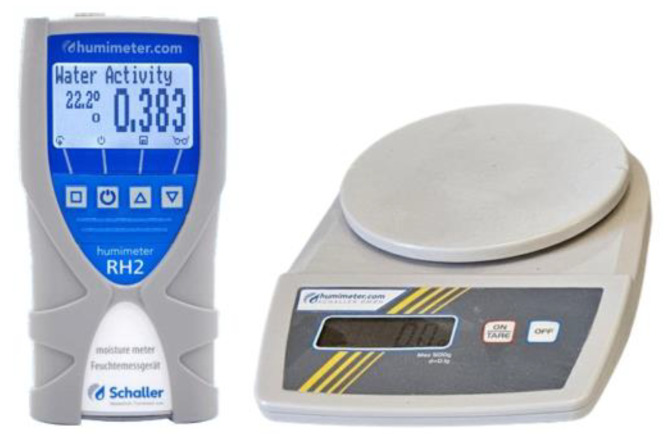
Schaller’s moisture meter (**left**) and scale (**right**).

The moisture content of the corn was analyzed at the inlet and outlet of the drying system. A Schaller portable moisture meter (Schaller GmbH, Gloggnitz, Austria), shown in Figure 2, was used for regular weighing and moisture measurements. During the moisture measurement, a sample of 300 g is taken. This quantity of grain is placed in a moisture meter, which can determine the corn moisture to one decimal place in a few seconds. To ensure accurate measurements, it is crucial that the corn to be measured is at the same temperature as the calibration temperature of the device.

**Figure 3:**
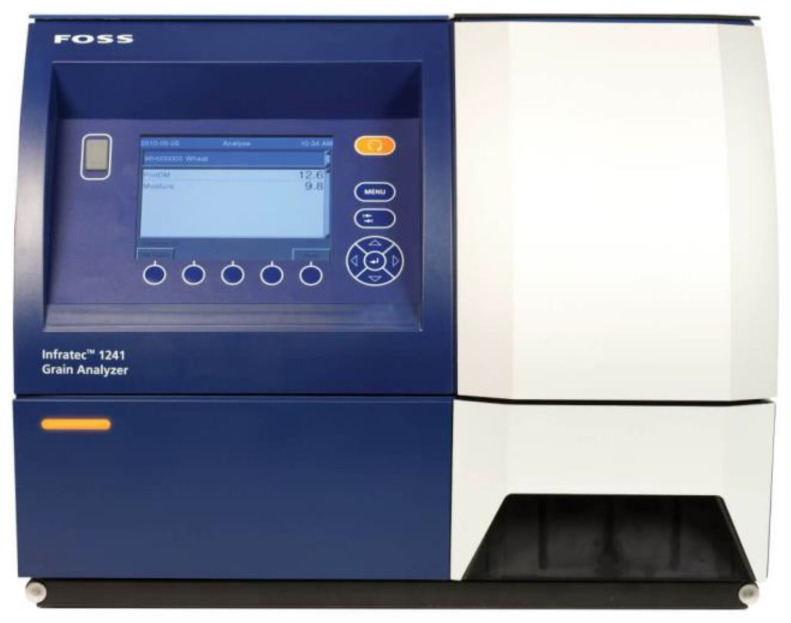
Foss Infratec grain analyzer.

To verify the accuracy of the measurements, systematic control was carried out using a more powerful laboratory device for grain analysis, the Infratec grain analyzer (Foss Analytical A/S, Hillerød, Denmark) [32] (Figure 3). This device is calibrated before each harvest season by an accredited Bureau Veritas laboratory (Bureau Veritas, Linhartova cesta 49A, 1000 Ljubljana, Slovenia) [33] and certified for different grain samples. This procedure ensures the appropriateness of the results. Like Schaller, the certified Infratec grain analyzer also provides moisture readings to one decimal place.

No major differences were found between the results obtained with the portable device and those obtained with the certified Infratec grain analyzer, although the results were slightly higher. The average absolute deviation between the two devices is 0.09% corn moisture. This confirms the high accuracy of the portable device and underlines the reliability of the results. The validation carried out ensured the quality and reliability of the data obtained when determining the moisture content of corn at the inlet and outlet of the drying system.

### 2.3. Weather Data Integration

In the dataset, seasonal variations in module temperatures were identified that affect the moisture content of the corn at the outlet. These fluctuations corresponded to changes in weather conditions, such as temperature, solar radiation, humidity, and precipitation. Consequently, understanding and controlling these changes proved to be essential for process optimization. Therefore, the dataset was supplemented with additional information from nearby weather stations called Radenci and Gačnik. The data were obtained from the website of the Environmental Agency of the Republic of Slovenia (ARSO). Two datasets were merged during the supplementation: the first contains the originally recorded data, and the second has columns with weather data. These data columns were combined based on the closest time interval.

### 2.4. Preparation of the Datasets

Due to the human-dependent nature of data collection, some values in the dataset were classified as missing. Therefore, a meaningful imputation of the data was necessary for efficient LSTM modeling. The percentage of missing data for each column is shown in Table 2.

#### Imputation Methods

To deal with missing values in the dataset, different imputation methods were applied based on the characteristics of the drying process. The chosen methods aimed to maintain consistency and temporal dependencies in the data.

1. Nearest neighbor imputation

Nearest neighbor imputation was performed using backward fill (bfill) and forward fill (ffill), which are mathematically defined as shown in Equations (1) and (2):

- Backward bfill:

*x_i_* = *x_j_* (1)

where *x_i_* is the missing value to be filled in, and *x_j_* is the next observed value in the dataset.

- Forward fill:

*x_i_* = *x_j_* (2)

where *x_i_* is the missing value to be replaced, and *x_j_* is the most recent observed value in the dataset that precedes *x_i_* [34].

Applied variables:

- Inlet corn moisture (*M_I_*): Filled with ffill and bfill, since the corn stored in the silo generally comes from the same batch and has a similar moisture content.
- Module temperatures (*T*_5_, *T*_6_): Process stability justifies ffill and bfill crediting.
- Material discharge interval (*D_I_*): Since the process was mainly regulated by *T_A_*, *D_I_* was mostly like its nearest neighbors.

2. Average neighbor imputation

This method was used when fluctuations in process control were present. The imputation of average neighbors can be described mathematically as shown in Equation (3):

(3) $$x_{i}=\frac{1}{\left|N_{i}\right|}\sum_{x_{j}\in N_{i}}x_{j}$$

where *x_i_* is the imputed value, |*N_i_*| is the number of neighboring data points for *x_i_*, and *x_j_* is each neighboring data point in *N_i_* [34].

Applied variable:

- Target air temperature (*T_A_*): Due to frequent process adjustments, a rolling window with 11 data points was used.

3. Linear interpolation

Linear interpolation was implemented using the following Equation (4) [35].

(4) $$f\left(x\right)=f\left(x_{0}\right)+\frac{f\left(x_{1}\right)-f\left(x_{0}\right)}{x_{1}-x_{0}}\left(x-x_{0}\right)$$

where *x* is the independent variable, *x*_1_ and *x*_0_ are known values of the independent variable, and *f*(*x*) is the value of the dependent variable for a value *x* of the independent variable.

Applied variable:

- Outlet corn moisture (*M_O_*): Based on the observed linear relationship.

4. Regression model

A regression model was implemented to estimate missing values in the *T*_3_ column. A significant part of the missing data in this column results from the additional installation of a sensor in drying module 3, as mentioned above. The model was trained on the recorded values of the module temperatures and other relevant parameters, using the linear relationships between the temperatures of the modules and other parameters. The regression equation is shown in Equation (5) [36].

(5) $$y=\beta_{0}+\beta_{1}x_{1}+\beta_{2}x_{2}+\cdots \beta_{n}x_{n}$$

where *y* is the dependent variable (*T*_3_), *x*_1_..., *x*_n_ are the independent variables (*T*_5_ and *T*_6_), and *β*_0_..., *β_n_* are the regression coefficients (weights). The regression coefficients for the specific variables of the third module are shown in Table 3.

Based on the regression training, the final equation for predicting the temperature of the third module (*T*_3_) is

(6) $$T_{3}=29.65+0.14T_{avg}+0.03{RH}_{avg}-0.43P-0.01{Rad}_{glob}-0.02M_{I}+0.045T_{A}+0.01T_{5}+0.174T_{6}-0.002D_{I}$$

The regression coefficients for the prediction of *T*_3_ were determined with a linear model fit, as shown in Table 2. The resulting Equation (6) replaces these estimated coefficients and can be used to replace missing *T_3_* values based on the correlated measurements from other modules and environmental conditions.

### 2.5. LSTM Implementation

A NumPy matrix was implemented for the data to enable LSTM modeling. The data were split into three subsets: training, validation, and test sets. The training set comprised 90% of the data, while the validation and test sets each comprised 5% of the data. To ensure the reliability of the results, normalization was performed to scale the data between 0 and 1 to minimize the effects of size differences between different features. A deep neural network, TensorFlow, was used to implement the LSTM model. In addition, a variety of hyperparameter combinations were investigated to optimize the LSTM model for predicting the moisture content of the corn at the exit of the drying system. The details of the hyperparameters investigated are listed in Table 4.

A model was used that follows a sequential approach. The first layer consisted of an LSTM unit containing 16,896 parameters. This was followed by three dense layers. The first and second dense layers contained 4160 parameters and the third 65 parameters. In total, the model had 25,281 parameters. All these parameters were trainable; i.e., they were adjusted during the training process.

## 3. Results and Discussion

The performance of the LSTM model in predicting the moisture content of dried corn was evaluated using different hyperparameter combinations. A total of 600 model configurations were tested, and the results were analyzed using standard performance metrics such as mean absolute error (MAE), root mean square error (RMSE), and mean squared error (MSE). In addition, 1200 visualizations were created to compare the actual and predicted moisture values.

Two datasets were used to assess the impact of missing data handling techniques:

- Imputed dataset—missing values were filled using imputation techniques.
- Alternative dataset—rows with missing values were removed.

In addition, a statistical analysis of the most important drying parameters was carried out to investigate how different weather conditions (temperature, humidity, precipitation, and solar radiation) influence the drying process. The results of this sensitivity analysis were visualized using a Pearson correlation heatmap to highlight significant relationships. This section is structured as follows:

- Section 3.1 gives a statistical overview of the dataset.
- Section 3.2 analyzes the effects of the weather variables on the performance of the drying system.
- Section 3.3 discusses the results of the model using the imputed dataset and highlights the hyperparameters that perform best.
- Section 3.4 compares the results obtained using the alternative dataset and assesses the impact of handling missing data on model accuracy.
- Section 3.5 outlines the limitations of the study, addressing factors such as data collection methods, environmental variability, and the generalizability of the model.

### 3.1. Statistical Presentation of the Dataset

Table 5 contains the descriptive statistics of the dataset, summarizing the most important process parameters recorded during the drying process. The table contains values for the mean, standard deviation, minimum, maximum, and percentile distributions of each variable, providing a general overview of their distribution and variability.

Important observations:

- The mean input moisture content (*M_I_*) was 25.4%, while the mean output moisture content (*M_O_*) was 13.6%, indicating an average moisture reduction of 11.8% during drying. However, the optimal moisture content for storage and processing is between 14% and 14.5%, indicating that the drying process can still be improved to better achieve the target moisture content.
- The discharge interval (*D_I_*) averaged 153.2 s, but with a wide range from 90 s to 600 s. The higher values (600 s) only occur during the start-up phase of the dryer when the system is warming up, resulting in temporary fluctuations in drying times before a stable operating state is reached.
- The temperature distribution between the drying modules (*T*_3_, *T*_5_, *T*_6_) shows relatively constant values, although *T*_5_ and *T*_6_ have higher temperatures than *T*_3_. This difference is due to their lower placement in the drying system, where the material remains longer in the dryer and allows for higher heat exposure before reaching the outlet.

### 3.2. Weather Variables’ Impact on Module Temperatures

**Figure 4:**
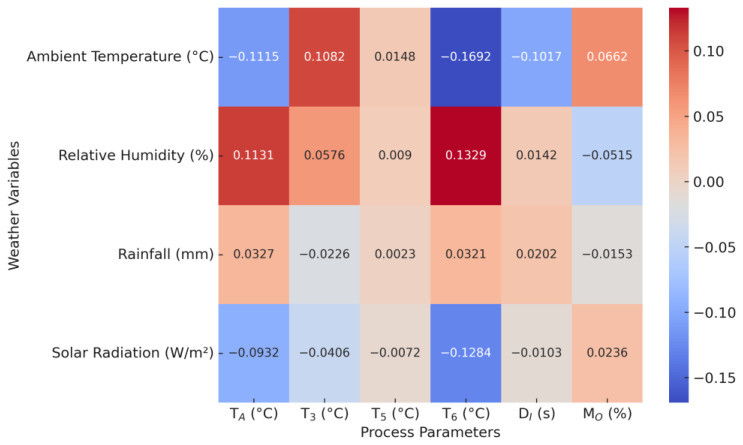
Sensitivity analysis: a heatmap of the Pearson correlation between weather data and process parameters.

Figure 4 shows a heatmap illustrating the Pearson correlations between weather variables and important process parameters.

1. Effects of ambient temperature on the drying system:
  - An increase in ambient temperature leads to a lower *T*_A_ (−0.1115). This means that operators can reduce the *T*_A_ when the ambient temperature is higher.
  - An increase in the ambient temperature leads to a higher *T_3_* (+0.1082).
  - An increase in the ambient temperature leads to a slightly higher *T*_5_ (+0.0148).
  - An increase in ambient temperature leads to a lower *T*_6_ (−0.1692), which is probably due to the reduction in *T*_A_ variables.
  - Increasing the ambient temperature allows users to reduce the *D*_I_ (−0.1017).

From examining the statistical data, we can conclude that changes in ambient temperature have noticeable effects on the process parameters, affecting *T_A_*, *T*_3_, *T*_5_, *T*_6_, and *D_I_*. However, the low correlation of *M_O_* (+0.0662) shows that the final moisture content remains largely unaffected. This suggests that operators are effectively adjusting drying conditions by, for example, lowering *T_A_* and *D_I_* to compensate for external temperature fluctuations.

2. Effects of relative humidity on the drying system:
  - An increase in relative humidity leads to higher *T_A_* settings (+0.1131).
  - An increase in relative humidity leads to a higher *T*_3_ (+0.0576).
  - An increase in relative humidity leads to a slightly higher *T*_5_ (+0.0090).
  - An increase in relative humidity leads to a higher *T*_6_ (+0.1329).
  - An increase in relative humidity leads to a slight increase in *D_I_* (+0.0142).
  - An increase in relative humidity leads to a lower *M_O_* (−0.0515).

These results show that, although relative humidity affects several process parameters, the influence is relatively small. The higher values of corn in the drying modules can be explained by higher *T_A_* settings. The inverse correlation with *M_O_* indicates that higher humidity does not significantly affect the final moisture content, which can be attributed to adjustments in the process settings.

3. Effects of precipitation on the drying system:
  - An increase in precipitation leads to a slight increase in *T_A_* (+0.0327), which indicates a small influence on the regulation of air temperature.
  - An increase in precipitation leads to a lower *T*_3_ (−0.0226), indicating that higher precipitation slightly reduces this process parameter.
  - An increase in precipitation leads to a negligible change in *T*_5_ (+0.0023), indicating that there is no significant influence.
  - An increase in precipitation leads to a slight increase in *T*_6_ (+0.0321), indicating that precipitation can slightly influence the heat exchange in the drying process.
  - An increase in precipitation leads to a slight increase in *D_I_* (+0.0202), indicating that an increase in precipitation may require a slight adjustment in drying intensity.
  - An increase in precipitation leads to a slight decrease in *M_O_* (−0.0153), indicating that precipitation has little direct influence on the final moisture content.

Despite these weak correlations, precipitation was included in the LSTM model to account for possible indirect and time-dependent effects. Precipitation can interact with humidity and temperature, leading to delayed effects on drying efficiency. In addition, the ability of the LSTM model to capture sequential dependencies allows the identification of relationships that may not be obvious in a static correlation analysis.

4. Effects of solar radiation on the drying system:
  - Higher solar radiation leads to lower *T*_A_ (−0.0932), suggesting that operators can reduce the need for a higher temperature setting due to external heat.
  - An increase in solar radiation leads to a lower *T*_3_ (−0.0406), indicating minimal direct influence on this process parameter.
  - An increase in solar radiation leads to a lower *T*_5_ (−0.0072), which indicates little to no direct influence.
  - Higher solar radiation leads to a lower *T*_6_ (−0.1284), indicating that higher solar radiation and other process adjustments reduce the energy required to maintain this temperature and therefore improve energy efficiency.
  - Higher solar radiation leads to a lower *D_I_* (−0.0103), indicating a slight reduction in the required drying intensity due to the additional heat input.
  - Higher solar radiation leads to a higher *M_O_* (+0.0236), indicating that higher solar radiation could slightly increase the final moisture content, although the effect remains minimal.

These results suggest that, while solar radiation has only a weak influence on individual process parameters, its broader role in changing environmental conditions may have a greater impact when analyzed in a time-dependent framework. The negative correlations of material temperatures inside the modules can be explained by a correlation with lower *T_A_* settings. Since solar radiation can contribute to nonlinear and lagged effects, its inclusion in the LSTM model remains crucial for capturing hidden patterns that are not obvious in the static correlation analysis.

### 3.3. Output of Algorithm on Imputed Dataset

From the 600 results obtained, the 10 best models with the lowest metric values were selected. In this way, useful hyperparameters for modeling the drying process of corn with LSTM neural networks were determined. The best combinations of hyperparameters for predicting outlet corn moisture content are listed in Table 6, with the optimal combination marked in bold.

The best configuration in our study, which uses the swish activation function, the Adam optimizer, a learning rate of 0.001, 16 epochs, and batch size none, achieves the lowest RMSE of 0.645, demonstrating the effectiveness of our chosen hyperparameters in minimizing prediction errors for moisture content in the drying process.

In comparison, the UVE-LSTM model developed by Xing et al. (2024) reported an RMSE of 0.711. The lower RMSE value of our model suggests a higher prediction accuracy in predicting the moisture content of corn at the output. This better performance can be attributed to our specific hyperparameter tuning and data preprocessing techniques, which optimized the model learning and reduced the generalization errors [38].

In contrast, Li et al. (2020) developed an intelligent artificial neural network model (IANN) to predict the moisture content (*M_C_*) in an industrial corn drying system. Their model achieved a mean square error (MSE) of 0.067 for *M_C_*, indicating a high prediction accuracy [39].

It is important to note that our study used practical data that were not originally intended for experimental purposes, whereas the study by Li et al. used data that were collected specifically for experimental studies. This difference in data collection methodology may affect the predictive accuracy of the models and their applicability in real-world scenarios. Our approach, using real-world data, shows potential for effective application in real-world industrial settings.

**Figure 5:**
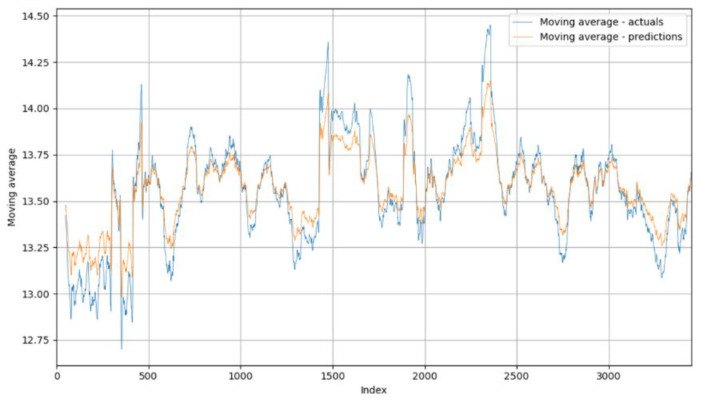
Moving averages of actual corn moisture measurements and predictions.

Moving averages were used to visualize the entire dataset, including actual samples and forecasts, to smooth out short-term fluctuations and highlight long-term trends or patterns in the data. The plot in Figure 5 shows a convincing match between the moving averages of the moisture samples (blue curve) and the moving averages of the forecasts (orange curve). This agreement means that the model adapts excellently to the actual corn moisture at the output. However, deviations from this occur, mainly with extreme measured values. These deviations are due to various factors originating in the actual process, such as malfunctions in the drying system, sudden changes in environmental conditions, and other failures of the drying system. In such cases, the model has difficulty in accurately predicting the outlet moisture content of the corn.

**Figure 6:**
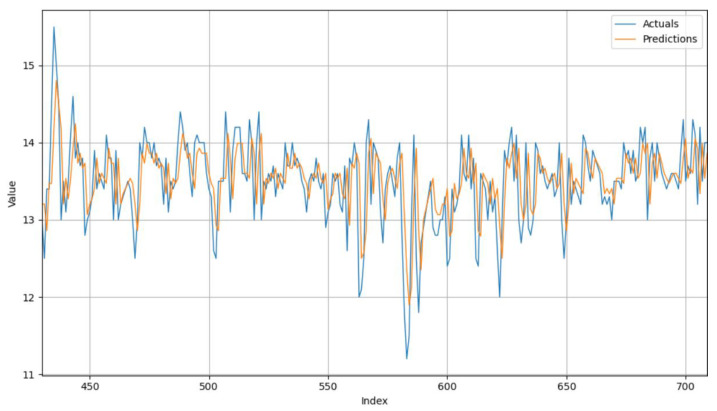
Visualization of samples and predictions in chosen interval.

To illustrate the actual corn moisture samples and the predictions, an interval was chosen in which the drying process ran smoothly and without failures or other disturbances. Specifically, samples from 430 to 710 were recorded, as shown in Figure 6. The blue curve represents the moisture content measurements at the outlet of the drying system, which fluctuate in a range from 11.2% to 15.5%. The predicted values (orange curve) also fluctuate in a similar range, namely, between 11.8% and 14.9%. These similarities show that the model effectively tracks changes in the moisture content of the corn at the exit.

### 3.4. Output of Algorithm on Alternative Dataset

As with the imputed dataset, the best combinations of algorithm outputs were also identified for the alternative dataset and are listed in Table 7. The combination of the ReLU activation function, the Nadam optimizer, a learning rate of 0.01, 64 epochs, and a batch size of 64 gave the best results, as shown by the minimum performance metrics, when the model was implemented on an alternative dataset. The best performing values are bolded in Table 7 for clarity. 

**Figure 7:**
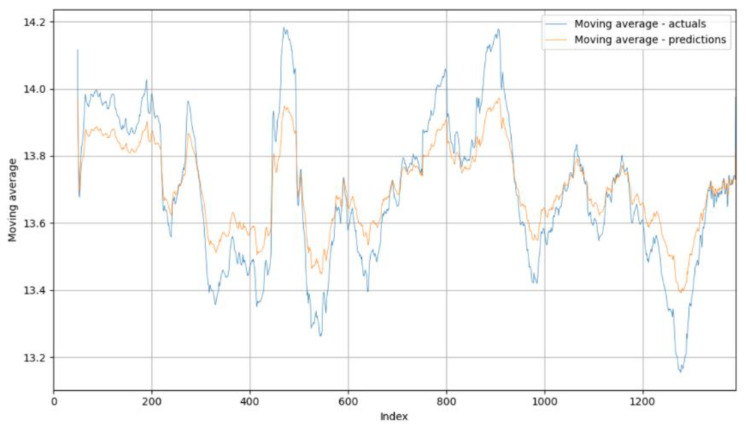
Moving averages of algorithm on alternative dataset.

The visualization of the moving averages was used to determine the quality of the algorithm implemented for the alternative dataset. The graph in Figure 7 illustrates the agreement between the moving averages of the moisture samples and the predictions. While the moving averages of the predictions generally follow the trends of the measurements, significant deviations can be seen in extreme situations. These deviations can be explained by the missing values in the sequences, which are crucial for LSTM modeling.

**Figure 8:**
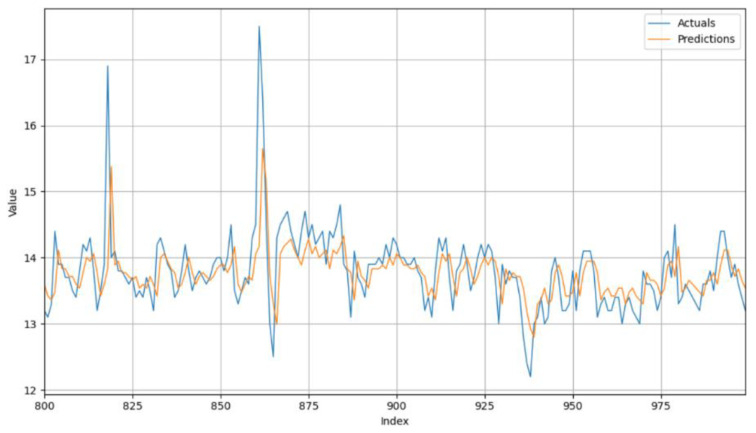
Visualization of the alternative dataset’s results in a specific interval.

To visualize the actual moisture measurements and the predictions, an interval between 800 and 100 was used based on the lowest number of missing values in the sequence. Figure 8 shows that the predictions are very close to the actual measurements. The moisture measurements vary between 12.2% and 17.5%. In this interval, the model accurately predicts fluctuations in corn moisture. The lowest predicted value is 12.8%, and the highest predicted value is 15.6%.

Based on the results of the algorithm implemented for both datasets, the use of imputation techniques was useful to ensure data consistency for LSTM modeling. Overall, the imputed dataset showed significantly better performance in predicting corn output moisture compared to the incomplete dataset with missing rows. However, it is important to note that other prediction techniques could be promising for predicting the target variable on an alternative dataset.

### 3.5. Study Limitations

The results show the effectiveness of the LSTM model in predicting outlet corn moisture content. However, some limitations should be considered to ensure a balanced interpretation of the results. These limitations mainly concern data availability, model assumptions, and external factors, all of which may affect the generalizability of the model.

1. Limited moisture measuring points and sampling intervals

Moisture content was measured at only two points—at the entrance and exit of the drying system—while three temperature values (*T*_3_, *T*_5_, and *T*_6_) were documented as indirect indicators of drying progress. In addition, moisture samples were taken at an almost fixed 1 h interval (±10 min), which limited the model’s ability to capture short-term fluctuations in drying behavior. Without real-time intermediate moisture measurements, the model assumes a uniform drying rate, which can lead to uncertainty in moisture predictions due to local drying variations, airflow fluctuations, and non-uniform heat distribution. Future improvements should incorporate real-time moisture sensors at multiple stages to increase model accuracy.

2. Effects of missing data and imputation techniques

Due to the human-dependent nature of data collection, there were missing values in the dataset that were addressed using imputation techniques. While these methods helped to maintain data consistency, the choice of imputation strategy could affect prediction accuracy by introducing estimation errors in key drying parameters.

○ Temperature values (*T*_3_, *T*_5_, *T*_6_):
  - The mean temperature of the drying modules ranged from 49.7 °C (*T*_3_) to 64.5 °C (*T_6_*), with a standard deviation of 2.15 °C across all modules.
  - Missing values in these parameters required imputation, which could result in an error of ±0.2 °C, estimated at 10% of the standard deviation (2.15 °C). Although this error is relatively small in the context of the overall temperature range (20 °C to 71 °C), it could still have a minor effect on the precise control of drying.
○ Moisture content (*M_I_*, *M_O_*):
  - The mean input moisture content (*M*_I_) was 25.4%, while the mean outlet moisture content (*M*_O_) was 13.6%, corresponding to an average moisture reduction of 11.8%.
  - The minimum recorded *M*_O_ was 10%, while the maximum reached 26%, with the peak occurring during the start-up phase, resulting in transient fluctuations in moisture content prior to stabilization.
  - Imputation of the missing moisture values could lead to a shift in model predictions, with potential errors estimated at ±0.3% based on the standard deviation of the recorded moisture values (0.8%). This could affect accuracy, especially near the thresholds for optimal corn storage (14% to 14.5%).
○ Discharge interval (*D_I_*):
  - *D*_I_ values range from 90 s to 600 s, with a mean of 153.2 s and a standard deviation of 40.7 s.
  - Missing *D_I_* values were imputed, resulting in an estimated uncertainty of ±15 s based on the standard deviation of the recorded values.
  - This uncertainty in the estimation of dwell time could have an impact on the prediction of drying efficiency, especially when optimizing the discharge timing for uniform moisture removal.

To minimize the errors associated with imputation, future work should focus on automatic data acquisition through sensor-based real-time recording systems.

3. Environmental variability and external conditions

The weather conditions were determined by two external weather stations located up to 10 km away from the drying plant. Due to spatial variability, differences between the recorded and actual conditions at the dryer site could affect the drying process. The following deviations were estimated based on typical local weather variability:

- Ambient temperature (°C): ±2.0 °C variation, affecting the drying air temperature and the moisture evaporation rate.
- Relative humidity (%): ±5.0% deviation, which affects the equilibrium moisture content of the corn and the overall drying efficiency.
- Precipitation (mm): ±1.5 mm variation, which can change the humidity conditions in the environment and affect the characteristics of the drying air.
- Solar radiation (W/m²): ±20 W/m^2^ variation, resulting in fluctuations in external heat input that can affect the required drying intensity.
- These discrepancies lead to potential variations in drying efficiency estimates and affect the prediction accuracy of the model in real applications. Future improvements should consider on-site environmental monitoring to minimize external measurement discrepancies.

These discrepancies lead to potential deviations in drying efficiency estimates and affect the predictive accuracy of the model in real-world applications. Future improvements should consider on-site environmental monitoring to minimize external measurement discrepancies.

4. Generalization and applicability to other drying systems

The model was developed using data from a single drying system with specific process conditions, which may limit its generalizability to other setups. While the methodology itself is adaptable, its effectiveness in different dryer designs, corn varieties, and operating conditions requires further validation. To ensure broader applicability, future work should include testing across multiple drying systems, diverse crop types, and varying environmental conditions to refine and improve model robustness.

5. The impact of the frequency of data collection on the performance of the model

The dataset consists of 3826 samples, but sampling intervals varied by 1 h (±10 min) due to manual data collection. This inconsistency may have affected the model’s ability to capture short-term drying trends and effectively learn time-dependent patterns.

To improve accuracy, future research should implement real-time sensor-based monitoring, ensure consistent data collection intervals, and apply interpolation techniques to compensate for past irregularities and improve model reliability.

6. The influence of the accuracy of the measuring devices

While the moisture content measurements were validated with the Infratec grain analyzer, the study mainly relied on a portable moisture meter (Schaller) for regular sampling. Despite its high accuracy, an average absolute deviation of 0.09% was found between the two devices. Although these differences are small, they could lead to slight deviations in the training data of the model. Future research should investigate the effects of using standardized, high-precision instruments for moisture measurements.

## 4. Conclusions

In this paper, a model was proposed to predict the moisture content of corn at the exit of the continuous drying system, depending on other process parameters of the system. The approach uses recurrent neural networks with long short-term memory (LSTM) architecture that considers multiple inputs to predict a single output: the moisture content of corn at the output. A method was proposed to use recorded analog data from an actual convective drying system for modeling. The algorithm utilized the recorded sequential data from the drying system to gain insight into the process and accurately predict the target variable. By combining advanced neural network techniques with real-world data, the approach provides a comprehensive framework for optimizing continuous drying systems in agricultural applications. The main conclusions of the study are as follows:

- The experimental approach of recording process parameters in addition to moisture measurements is critical to accurately predicting the initial moisture content of corn in continuous drying systems.
- By applying imputation techniques, it is possible to prepare a human-dependent dataset for efficient modeling with neural networks.
- LSTM neural networks have proven to be a highly effective method for predicting moisture content based on key variables, including the target air temperature (*T_A_*), material discharge interval (*D*_I_), inlet moisture content, drying module temperatures (*T*_3_, *T*_5_, *T*_6_), and environmental conditions.
- The performance of the model was evaluated using various statistical metrics: mean square error (MSE), root mean square error (RMSE), mean absolute error (MAE), and mean absolute percentage error (MAPE). The results indicate excellent predictive accuracy, as evidenced by the following metrics: RMSE = 0.645, MSE = 0.416, MAE = 0.352, and MAPE = 2.555.

The research also contributes to the sustainability of the drying process. The proposed LSTM model for predicting the moisture content of corn at the outlet has demonstrated its ability to estimate moisture content based on key process parameters, providing a basis for potential improvements in process efficiency:

- The target air temperature can be minimized, resulting in lower energy consumption, so that fewer natural resources are used.
- The material discharge intervals can be adjusted as required so that drying times are set to the optimal conditions and more of the product can be processed.
- The desired moisture content at the outlet of the drying system results in a greater mass and higher quality of the product.

Currently, the model is used for the offline analysis of drying process data, allowing operators to evaluate trends in moisture predictions, analyze past drying performance, and estimate the optimal settings for *T_A_* and *D_I_* based on the model’s predictions. This enables a data-driven approach to refining drying parameters over time. However, the model has not yet been integrated into an automated control system for real-time process adjustments.

Further research is required to develop a fully automated system where the real-time prediction of moisture content would dynamically adjust independent variables such as the target air temperature (*T*_A_) and material discharge interval (*D*_I_). Furthermore, future work should investigate the inclusion of additional system parameters, such as air velocity, ambient humidity, and energy consumption, to improve the robustness of the model and its applicability under different drying conditions.

The results of this study indicate that LSTM-based modeling has significant potential for improving the control of drying processes. However, further validation and implementation steps are required before it can be used in real-time industrial environments.

Future research is needed to develop automated data acquisition systems that do not rely on human input, particularly in the context of recording process parameter data and moisture samples. Other methods for sequential data modeling, such as temporal convolutional neural networks (TCNs) and gated recurrent units (GRUs), can also be tested in the future.

## Figures and Tables

**Table 1:** Drying conditions of the continuous drying system.

Parameter	Value/Range	Description
Drying process	Continuous Convective	Downward material flow through drying modules
Material flow rate	9–12 t/h	Controlled by discharge interval
Discharge interval (*D_I_*)	Adjustable (120–160 s, avg: 153.2 s)	Regulates corn retention time
Target air temperature (*T_A_*)	Adjustable (Max: 120 °C)	Higher *T_A_* speeds up drying but is limited to avoid grain damage
Module temperatures (mean)	*T*_3_: 49.7 °C, *T*_5_: 59.5 °C, *T*_6_: 64.5 °C	Heat distribution across drying tower
Inlet moisture (*M_I_*)	25.4% (mean)	Mean moisture value before drying
Outlet moisture (*M_O_*)	13.6% (mean)	Mean moisture value after drying

**Table 2:** Percentage of missing data for each column.

Column	*M_I_*	*T_A_*	*T*_3_	*T*_5_	*T*_6_	*D_I_*	*M_O_*
Missing data (%)	5.18	0.78	28.94	1.07	1.12	0.97	1.86

**Table 3:** Regression coefficients for module 3 imputation.

**Label**	**Symbol in Equation**	**Coefficient/Value**
Intercept ()	*β*_0_	29.65
Average air temperature—β_1_	*T_avg_* (*β*_1_)	0.14
Average relative humidity (%)	*RHavg* (*β*_2_)	0.03
Precipitation (mm)	*P* (*β*_3_)	−0.43
Global radiation (W/m^2^)	*Rad_glob_* (*β*_4_)	−0.01
Inlet moisture	*M_I_* (*β*_5_)	−0.02
Target air temperature	*T_A_* (*β*_6_)	0.045
Module 5 temperature	*T_5_* (*β*_7_)	0.01
Module 6 temperature	*T_6_* (*β*_8_)	0.174
Material discharge interval	*M_D_* (*β*_9_)	−0.002

**Table 4:** Tested hyperparameters.

Optimizers	Adam, Adagrad, Ftrl, Adadelta, Nadam
Activations	Relu, Prelu, Elu, Selu, Swish
Learning rates	0.01, 0.001
Epochs	16, 64, 100
Batch sizes	None, 16, 32, 64

**Table 5:** Statistical representation of the dataset.

	*M_I_*	*T_A_*	*T*_3_	*T*_5_	*T*_6_	*D_I_*	*M_O_*
Mean	25.4	111.7	49.7	59.5	64.5	153.2	13.6
Std Dev	2.5	7.4	2.15	3.61	4.1	40.7	0.8
Min	14.0	60.0	20.0	19.0	18.0	90.0	10.0
25th Percentile	23.3	110	48.9	58.0	63.0	135.0	13.2
50th percentile	25.3	110	49.8	60.0	65.0	145.0	13.6
75th Percentile	26.7	118	50.6	61.0	67.0	160.0	13.9
Max	36.8	125	71.0	82.0	85.0	600.0	26.0

**Table 6:** Best hyperparameter combinations for corn moisture prediction calculated on imputed dataset [37].

Activation	Optimizer	Learning Rate	Epochs	Batch Size	MSE	RMSE	MAE	MAPE
PReLU	Adam	0.001	16	32	0.418	0.647	0.352	2.554
elu	Adam	0.001	16	16	0.418	0.647	0.352	2.553
elu	Adagrad	0.001	100	64	0.419	0.647	0.352	2.555
elu	Adadelta	0.001	64	32	0.419	0.647	0.352	2.555
elu	Adadelta	0.001	64	64	0.418	0.647	0.352	2.557
elu	Nadam	0.001	16	16	0.418	0.647	0.352	2.554
elu	Ftrl	0.001	64	16	0.419	0.647	0.352	2.558
swish	Ftrl	0.01	16	32	0.417	0.646	0.352	2.552
**swish**	**Adam**	**0.001**	**16**	**None**	**0.416**	**0.645**	**0.352**	**2.555**
swish	Nadam	0.001	16	None	0.419	0.647	0.352	2.553

**Table 7:** Best hyperparameter combinations for outlet corn moisture prediction calculated on alternative dataset.

Activation	Optimizer	Learning Rate	Epochs	Batch Size	MSE	RMSE	MAE	MAPE
Relu	Adam	001	16	32	12.38	3.52	0.428	2.49
Relu	Adam	0.01	64	64	12.37	3.52	0.432	2.53
Relu	Nadam	0.01	16	64	12.46	3.53	0.432	2.51
**Relu**	**Nadam**	**0.01**	**64**	**64**	**12.37**	**3.52**	**0.428**	**2.49**
PReLU	Ftrl	0.01	64	32	12.35	3.51	0.439	2.58
PReLU	Ftrl	0.01	100	16	12.34	3.51	0.439	2.58
Elu	Nadam	0.001	64	None	12.45	3.53	0.433	2.52
Selu	Adagard	0.01	16	16	12.48	3.53	0.429	2.46
Selu	Ftrl	0.01	16	16	12.5	3.54	0.436	2.54
Selu	Nadam	0.001	100	16	12.45	3.53	0.43	2.5

## Data Availability

The original contributions presented in the study are included in the article, further inquiries can be directed to the corresponding author.
